# Antifungal Susceptibility and Biofilm Production of *Candida* spp. Isolated from Clinical Samples

**DOI:** 10.1155/2018/7495218

**Published:** 2018-10-10

**Authors:** Munmun B. Marak, Biranthabail Dhanashree

**Affiliations:** Department of Microbiology, Kasturba Medical College, Mangalore, Manipal Academy of Higher Education, Manipal, India

## Abstract

**Objective:**

The study aims to speciate clinical *Candida* isolates and detect their biofilm-forming ability and antifungal resistance.

**Methods:**

All the *Candida* spp. isolated from different clinical samples like pus, urine, blood, and body fluid were included in the study. Biofilm production was tested by the microtiter plate method. Antifungal susceptibility was studied by the disk diffusion method. Patient's demographic details such as age, sex, and clinical information were collected. Presence of other risk factors such as diabetes mellitus, history of antibiotic use, and any urinary tract instrumentations was also recorded.

**Results:**

Among 90 *Candida* species isolated, most predominant species was found to be *C. albicans* (45.5%) followed by *C. tropicalis* (28.88%), *C. krusei* (20%), *C. glabrata* (3.33%), and *C. parapsilosis* (2.22%). *Candida* spp. were isolated from urine (43%), BAL/sputum (18.88%), high vaginal swab (8.88%), suction tips (7.77%), blood and wound swabs (6.66%), pus (3.33%), bile aspirate (2.22%), and deep tissue (1.11%). A larger number of females were affected than males, and the age group of 51 to 60 years was more susceptible to candidiasis. A higher number of *C. albicans* isolates produced biofilm followed by *C. parapsilosis*, *C. tropicalis*, and *C. krusei.* However, *C. glabrata* showed no biofilm production in our study. All *Candida* isolates were 100% sensitive to amphotericin B. Voriconazole was the next effective drug with 81.11% susceptibility. 24.44% of strains were resistant to fluconazole.

**Conclusion:**

Speciation of *Candida* isolates, detection of ability to form the biofilm, and monitoring of antifungal susceptibility testing are necessary for appropriate treatment.

## 1. Introduction

Recent advances in research technology have allowed researchers to study bacteria and fungi in their natural environment, and over 95% of bacteria existing in nature are in biofilms [1]. *Candida* species are found as normal flora in healthy individuals and are known to cause opportunistic infections with high rates of mortality, especially in immunocompromised individuals [2]. *Candida* spp. cause systemic diseases which are the fourth leading cause of nosocomial bloodstream infections in modern hospitals. The most challenging clinical problem is the increased rate of non-*Candida albicans* isolation and the rapidly growing resistance of *Candida* species [3].

*Candida albicans* is the most prevalent among *Candida* spp., which causes both superficial and systemic infections. Other pathogenic *Candida* species include *C. tropicalis*, *C. glabrata*, *C*. *parapsilosis*, and *C. krusei* accounting for 25%, 8%, 7%, and 4% of candidiasis, respectively [4]. Pathogenesis of candidiasis depends on the expression of virulence factors like germ tube formation, adhesions, phenotypic switching, biofilm formation, and the production of hydrolytic enzymes [5].

A majority of the diseases caused by *Candida* spp. are due to biofilm formation. Biofilms are the group of microorganisms that are embedded in an extracellular matrix (ECM), forming a complex three-dimensional architecture on biotic and abiotic surfaces [6]. Biofilm formation can occur on mucosal surfaces and plastic surfaces of indwelling devices. Biofilms are genetically resistant to antifungal agents including amphotericin B (AMB) and fluconazole (FLU). Biofilm formation varies depending on the *Candida* spp. [7]. Most frequently, pathogenic effects are caused by *Candida albicans* and to a lesser extent by other *Candida* spp. [8].

*Candida* shows resistance to azole due to general and long-term use of it [9]. Candidiasis can reoccur repeatedly. Some health-care providers prescribe antifungal drugs on a long-term basis, but this can lead to drug-resistant candidiasis that is more difficult to treat. Therefore, early identification of *Candida* spp. and monitoring their antifungal susceptibility help in treatment. Since there are very few studies on biofilm formation and drug resistance reported from India, the present study is undertaken to isolate *Candida* species from various clinical specimens, detect biofilm formation, and to study their antifungal susceptibility pattern.

## 2. Materials and Methods

### 2.1. Study Population

*Candida* species (*n* = 90) isolated from clinical samples like blood, urine, body fluids, and pus, received at the Department of Microbiology, Kasturba Medical College (KMC), Mangalore, was included in the study after obtaining ethical clearance from the Institutional Ethics Committee. The genus *Candida* was identified by colony morphology, Gram-staining, and other standard biochemical reactions [10]. All the media and chemicals used in the study were procured from Hi-Media Laboratories Pvt. Ltd., Mumbai, India.

### 2.2. Data Collection

Patient's demographic details such as age, sex, and clinical information were collected. Presence of other risk factors such as diabetes mellitus, history of antibiotic use, and any urinary tract instrumentations was also recorded from the hospital information system.

### 2.3. Antifungal Susceptibility Testing

Fungal susceptibility to routinely used drugs like amphotericin B (100 units), fluconazole (25 *μ*g), and voriconazole (1 *μ*g) was done by the disk diffusion method, using Mueller-Hinton agar supplemented with 0.5 mg/ml methylene blue. Agar plates were inoculated with a suspension of yeast cells whose turbidity was adjusted to 0.5 McFarland standards (10^6^ CFU/ml) in a manner that is currently being used for testing antibacterial agents. Antifungal disks were placed on the inoculated plates and incubated at 27°C for 24–48 hours. The diameter of the zone of inhibition was measured. Results were interpreted as per CLSI guidelines [11, 12].

### 2.4. Biofilm Production by Microtiter Plate Method (MTP)

Biofilm formation was performed on a sterile 96-well microtiter plate. A colony of each isolate was inoculated into tubes containing two ml of brain heart infusion broth (BHIB) and incubated at 37°C for 24 hours. All the broth cultures were diluted at a ratio of 1 : 20 using fresh BHIB, and 200 *μ*l was placed into microtiter plates and incubated at 37°C for 24 hours. After the completion of incubation, microtiter plates were emptied, rinsed with distilled water three times, and then inverted to blot. Each well was then filled with 200 *μ*l of 1% crystal violet and incubated for 15 mins. After incubation, the microplates were again rinsed three times with distilled water. Then 200 *μ*l of ethanol: acetone mixture (80 : 20 w/v) was added to each well and were read at 450 nm using an ELISA reader, and OD was recorded for each well. Sterile BHIB without microorganisms was used as the negative control. The cut-off value was determined by arithmetically averaging the OD of the wells containing sterile BHIB and by adding a standard deviation of +2. Samples with an OD higher than the cut-off value were considered positive, whereas those with the lower optical density than the cut-off were deemed to be negative [13].

## 3. Results

In the present study, 90 *Candida* spp. were isolated from different clinical samples, which included *C. albicans* (45.5%) followed by *C. tropicalis* (28.88%), *C. krusei* (20%), *C. glabrata* (3.33%), and *C. parapsilosis* (2.22%). They were subjected to antifungal susceptibility testing and biofilm formation. Results are summarized as frequency tables, and percentages were worked out. Statistical analysis was performed by using Statistical Package SPSS V.16.0. Proportions and association were calculated by the descriptive study test and unpaired Student's *t*-test.

Table 1 shows various *Candida* spp. isolated from clinical specimens. In the present study, 41 *Candida* spp. were obtained from males and 49 from females as shown in Table 2.

Clinical history was obtained from the medical records. Among them, 25 patients had diabetes mellitus, nine were catheterized, 21 had signs and symptoms of UTI, four were cancer patients, eight were pregnant women, 14 with chronic kidney disease, 17 with a respiratory infection, and one had heart disease.

Out of 41 *Candida albicans* isolates, 21 (51.2%) strains were positive for biofilm production.

Among the total 49 non-*Candida albicans*, 28 (57.14%) isolates were biofilm producers (Table 3).

Antifungal susceptibility testing of various *Candida* species showed that all isolates were susceptible to amphotericin B. The result of antifungal susceptibility of different *Candida* spp. is shown in Table 4.

## 4. Discussion

*Candida* spp. is found to be commensal, and interruption of standard host defense is required for them to act as pathogens. As there is an increase in the number of patients who are immunocompromised, aged, receiving antibacterial and aggressive cancer chemotherapy, or undergoing invasive surgical procedures and organ transplantation, Candidiasis has emerged as an alarming opportunistic disease [14].

The present study showed a significant variation in the distribution of *Candida* spp in different clinical samples. The most predominant species was found to be *C. albicans* (45.5%), followed by *C. tropicalis* (28.88%), *C. krusei* (20%), *C. glabrata* (3.33%), and *C. parapsilosis* (2.22%), respectively. Our findings are similar to the previously reported data by Sajjan et al. [15]. Zarei-Mahmoudabadi et al. and Chakrabarti et al., however, reported a lower prevalence rate (39% and 25%) of *C. albicans* [16, 17].

In the present study, highest recovery of *Candida* spp. was from urine (43%) followed by BAL/Sputum (18.88%), high vaginal swab (8.88%), central line/ET/oral suction tip (7.77%), blood and wound swabs (6.66%), pus (3.33%), bile aspirate (2.22%), and deep tissue and skin scrapping (1.11%), respectively. However, the study done by Saijan et al. (2014) showed the highest recovery of *Candida* spp from high vaginal swabs [15]. This could be because, in our study, we have processed more number of urine samples than the high vaginal swabs.

In the present study, it is found that candidiasis can occur at all ages and both sexes. The youngest being a one-year-old infant, while the oldest was 88 years. According to surveys conducted by Emeribe et al. [18] and Puri et al. [19]*Candida* infection was found the maximum in the age group of 21–40 years. In our study, maximum isolation of *Candida* spp was found in the age group of 51 to 60 years. Higher susceptibility of older adults to *Candida* infection could be due to lower immunity, aging, predisposing factors such as diabetes mellitus and malignancies. More number of females were affected than males with an incidence of 49 (54%) and 41 (45%), respectively, which is similar to the study done by earlier workers. The rate of isolation of *Candida* spp. in females was about 61.2%, while in males, it was only 38% [15]. In the present study, the distribution of *Candida* spp. among the males and females was found to be similar.

One of the most frequent predisposing factors in our study was diabetes mellitus which accounted for 28% of the total cases, followed by patients with symptomatic urinary tract infection (24%), pregnancy (8.8%), and catheterized chronic kidney disease (15.5%), respectively. However, Arora et al. demonstrated intravenous cannula (63%) followed by prolonged use of antibiotics (35%) and immunosuppression (23%) to be the most common risk factors in their study [20]. The risk factors may vary from geographical areas and may also be due to underlying conditions and food habits/immunity of patients studied.

Biofilms are universal, complex, interdependent communities of surface-associated microorganisms, enclosed in an exopolysaccharide matrix occurring on any surface, including medical devices [21]. The pathogenicity of *Candida* species is associated with its ability to form Biofilm and is an essential virulence determinant during candidiasis [14]. The present study showed biofilm production to be 57.14% among non-*C. albicans* spp. and 39.02% among *C. albicans*. This result is nearly similar to those reported by Kumar et al. [22].

Among the non-*Candida albicans* spp. studied for biofilm production in the present study, 100% of *C. parapsilosis* showed biofilm formation, followed by *C. tropicalis* (61.53%) and *C*. *krusei* (55.55%). However, Kuhn et al. showed that *C. albicans* produces quantitatively more biofilms than other *Candida* species [23]. This difference could be due to the usage of different methods like silicone elastomer for biofilm production and tetrazolium for quantification. However, in the present study, the microtiter plate method with crystal violet stain was used. *C. glabrata* did not show biofilm production in our study. The chi-square test showed a nonsignificant variation in the number of biofilm producers and nonproducers.

It is crucial to monitor antifungal resistance among *Candida s*pecies because it gives clues to emerging threats of new resistant strains that help in empirical treatment. Among the 90 *Candida* isolates tested, all were susceptible to amphotericin B in our study which is similar to the results reported by Arora et al. [24]. Therefore, *Candida* strains isolated in our study area have not yet developed resistance to amphotericin B.

75.55% strains were sensitive to fluconazole, and 24.44% were resistant. Among the nonsusceptible *Candida* spp. 40.90% were *C. albicans*, 36.36% were *C. tropicalis,* and 22.72% were *C. krusei*. 18.88% of the *Candida* spp. were resistant to voriconazole, among which 17.64% were *C. krusei* and 41.17% were *C. albicans* and *C. tropicalis*. However, Yenisehirli et al. reported 34% and 14% resistance rates of fluconazole and voriconazole among *C. albicans*, respectively [25]. Jayalaksmi et al. published a resistance rate of 34.3% to fluconazole among 105 *Candida* isolates recovered from different clinical specimens [26]. A study conducted by Pelletier et al. revealed that 42 out of 295 *Candida* isolates were showing reduced susceptibility to fluconazole [27]. Our resistance rates of fluconazole and voriconazole are in line with those of earlier studies. The possibility of reduced susceptibility to fluconazole and voriconazole may be due to widespread and long-term use of those antifungals among the study subjects. There is no statistical correlation between the biofilm formation and antifungal susceptibility (*p* > 0.05).

## 5. Conclusion

*Candida* infection depends on the underlying conditions of the patients. Antifungal resistance is emerging in our *Candida* isolates. However, there was no correlation between biofilm formation and antifungal resistance among our candidal strains.

## Figures and Tables

**Table 1:** *Candida* spp. isolated from various clinical samples.

Type of clinical specimen	*C. albicans* (*n*)	Non-*Candida albicans* spp. (*n*)	Total *Candida* isolates (*n*)
Blood	1	5	6
Aspirated pus	1	2	3
BAL/sputum	13	4	17
Urine	14	25	39
Central line/ET/oral			
Suction tip	2	5	7
Bile aspirate	0	2	2
High vaginal swab	7	1	8
Deep tissue	0	1	1
Wound swabs	3	3	6
Skin scraping	0	1	1
Total	41	49	90

**Table 2:** Age-wise and sex-wise distribution of *Candida* spp.

Age group	Sex		*Candida* isolates					Total (*n*)
	Male	Female	*C. albicans*	*C. tropicalis*	*C. krusei*	*C. glabrata*	*C. parapsilosis*	
0–10	—	1	1	—	—	—	—	1
11–20	1	3	2	1	1	—	—	4
21–30	—	7	5	—	2	—	—	7
31–40	3	5	3	2	2	1	—	8
41–50	6	6	4	1	6	1	—	12
51–60	13	12	11	9	4	1	—	25
61–70	6	8	6	5	1	—	2	14
71–80	10	4	7	6	1	—	—	14
81–90	2	3	2	2	1	—	—	5
Total	41	49	41	26	18	3	2	90

**Table 3:** Biofilm formation by various *Candida* species.

*Candida* species	Biofilm negative	Biofilm positive	Total
*C. albicans*	20	21	41
*C. tropicalis*	11	15	26
*C. krusei*	7	11	18
*C. glabrata*	3	0	3
*C. parapsilosis*	0	2	2
TOTAL	41	49	90

**Table 4:** Antifungal susceptibility testing of *Candida* species.

Antifungal agents		*Candida* species					Total (*N* = 90)
		*C. albicans* (*N* = 41)	*C. tropicalis* (*N* = 26)	*C. krusei* (*N* = 18)	*C. glabrata* (*N* = 3)	*C. parapsilosis* (*N* = 2)	
Amphotericin B	S	41 (100%)	26 (100%)	18 (100%)	3 (100%)	2 (100%)	90 (100%)
	R	—	—	—	—	—	—
Fluconazole	S	32 (47.05%)	18 (26.47%)	13 (19.11%)	3 (100%)	2 (100%)	68 (75.55%)
	R	9 (40.90%)	8 (36.36%)	5 (22.72%)	—	—	22 (24.44%)
Voriconazole	S	34 (46.57%)	19 (26.02%)	15 (20.54%)	3 (100%)	2 (100%)	73 (81.11%)
	R	7 (41.17%)	7 (41.17%)	3 (17.64%)	—	—	17 (18.88%)

S = susceptible; R = resistant.

## Data Availability

The experimental data used to support the findings of this study are available from the corresponding author upon request.
